# Expression of Cartilage Oligomeric Matrix Protein in colorectal cancer is an adverse prognostic factor and correlates negatively with infiltrating immune cells and PD-L1 expression

**DOI:** 10.3389/fimmu.2023.1167659

**Published:** 2023-05-03

**Authors:** Anna M. Blom, Chrysostomi Gialeli, Catharina Hagerling, Jonna Berntsson, Karin Jirström, Konstantinos S. Papadakos

**Affiliations:** ^1^ Division of Medical Protein Chemistry, Department of Translational Medicine, Lund University, Malmö, Sweden; ^2^ Cardiovascular Research - Translational Studies, Department of Clinical Sciences, Lund University, Malmö, Sweden; ^3^ Division of Translational Cancer Research, Department of Laboratory Medicine, Lund University, Lund, Sweden; ^4^ Oncology and Therapeutic Pathology, Department of Clinical Sciences Lund, Lund University, Lund, Sweden

**Keywords:** colon cancer, T-cells, PD-L1, collagen, fibrosis, Cartilage Oligomeric Matrix Protein (COMP)

## Abstract

**Introduction:**

Cartilage Oligomeric Matrix Protein (COMP) is an oncogenic protein that has been associated with a decrease in infiltrating T-cells in periampullary adenocarcinoma. This study aimed to investigate whether this is also the case for colorectal cancer (CRC) and to evaluate the relationship between COMP expression and clinopathological features.

**Methods:**

Immunohistochemistry was used to determine the expression levels of COMP in tumor cells and stroma in primary tumors from a cohort of 537 CRC patients. The expression of immune cell markers, including CD3+, CD8+, FoxP3+, CD68+, CD56+, CD163+, and PD-L1, was evaluated previously. Tumor fibrosis was assessed by Sirius Red staining and evaluation of collagen fiber organization.

**Results:**

COMP expression correlated positively with TNM-stage and grade of differentiation. Patients with CRC expressing high levels of COMP had significantly shorter OS than those with low COMP expression (p<0.0001), and fewer infiltrating T-cells were detected in tumors with high COMP expression. Additionally, a negative correlation was identified between the expression of COMP and PD-L1 on both tumor cells and immune cells. Cox regression analysis showed that tumors expressing high levels of COMP had significantly shorter OS, independent of all evaluated immune cell markers. Tumor fibrosis was correlated with high expression of COMP in the stroma (p<0.0001), and tumors with high levels of COMP expression and denser fibrosis displayed more sparse immune cell infiltration.

**Discussion:**

The results suggest that COMP expression in CRC may exert an immune regulatory effect by increasing dense fibrosis and decreasing immune cell infiltration. These findings support the notion that COMP is an important factor in the development and progression of CRC.

## Introduction

Colorectal cancer (CRC) is the third most commonly diagnosed cancer (10.0%) and the third leading cause of cancer death worldwide (9.4%) (1). Early detection followed by surgical excision and neoadjuvant therapy are crucial for a favorable outcome. Tumor stage at diagnosis is still the most important prognostic factor and it would be advantageous to discover further biomarkers to identify high-risk disease and potential resistance to treatment. According to consensus, CRC classification is centered around the gene expression pattern and categorized into four molecular subtypes (2). CMS1 includes tumors with microsatellite instability (MSI) and gene expression signature related to the immune system. CMS2 is considered the most canonical and is associated with mutations in *APC*, *p53* and *RAS* genes. Also, the epidermal growth factor receptor (EGFR) pathway is activated as a result of overexpression of EGFR-ligands (amphiregulin, epiregulin and preferred dimerization partner of EGFR, the epidermal growth factor receptor 2. CMS3 is characterized by metabolic imbalance with enhanced glutaminolysis and lipidogenesis. CSM4 is the most chemoresistant subgroup, with enhanced expression of the transforming growth factor beta (TGF-β) pathway and induction of epithelial to mesenchymal transition (EMT).

Recent studies of the tumor microenvironment revealed the significance of the crosstalk between the extracellular matrix (ECM), cancer-associated fibroblasts (CAFs), and infiltrating immune cells (3). Cartilage oligomeric matrix protein (COMP) is a pentameric protein almost exclusively expressed in cartilage, where it was extensively studied as a molecule contributing to collagen secretion and cartilage ECM organization. The levels of COMP in sera can be used as a marker of cartilage turnover (4). Unexpectedly, we detected COMP expression in tumors from patients with breast cancer (5). COMP expression by the cancer cells (5), as well as the levels of COMP in the serum of patients with breast cancer (6) were an independent prognostic factor of patient survival. *In vivo* xenograft models revealed that mice orthotopically inoculated with COMP-expressing breast cancer cells developed larger tumors, and more frequent metastases in the lymph nodes and the lungs compared to mice inoculated with wild-type cells. Moreover, breast cancer cell lines expressing COMP were more invasive, less prone to apoptosis, and had enhanced Warburg metabolic effect (5). The underlying molecular mechanism involves the activation of Notch3 by its ligand Jagged1 bridged together by COMP, which leads to a generation of a higher proportion of cancer stem cells (7).

An important role of COMP in tumor progression was also revealed in prostate cancer (8), hepatocellular carcinoma (9), urothelial cancer (10), and CRC (11). CRC patients with tumors expressing COMP mRNA had reduced overall survival (OS) and recurrence-free survival (RFS). The expression of COMP correlated with TNM-classification of the disease and high grade of differentiation. The serum levels of COMP were elevated in CRC patients compared with healthy patients and curative resection of COMP-expressing tumors reduced the serum levels of COMP. When the *COMP* gene was knocked out in LoVo CRC cells, the proliferation of cells was reduced, and in a xenograft mouse model, LoVo-COMP knockout cells formed smaller tumors than the control cells. The underlying mechanism was hypothesized to include the AKT-mTOR pathway (11). Further, COMP interacts with actin-binding protein transgelin, which increases cell migration and epithelial to mesenchymal transition (12). Subsequent studies confirmed the epidemiological findings of the study (13–16), but the underlying molecular mechanism needs to be fully established in the future.

COMP is amongst the most upregulated proteins in idiopathic pulmonary fibrosis, a chronic inflammatory lung disease (17, 18). Also, in scleroderma, high levels of fibrosis correlate with elevated levels of COMP expression (19). Under fibrotic conditions, COMP expression may be initiated by TGF-β in skin fibroblasts and lung epithelial cells. After this first step in which COMP expression is triggered, a vicious cycle is initiated whereby COMP stimulates TGF-β activity, which increases the expression of COMP (19, 20) ultimately leading to stiffer fibrosis. Moreover, COMP increases the expression of type I collagen and facilitates its secretion, acting as an intracellular chaperon (20, 21). Mice knocked-out for COMP expression are not able to deposit collagens type I and XII in fibrotic tissue. The induced fibrosis in these mice was characterized by reduced fibrotic markers and impaired ability to assembly collagen fibers (21).

Investigating the expression of COMP in patients with periampullary adenocarcinoma, we discovered that T-cells were excluded from the cancer cell compartment in tumors with high levels of COMP expression (22). The phenomenon could be partially explained by the elevated fibrosis, which was detected by evaluating the amount and organization of collagen in the tumors (22). COMP is already known to be expressed in CRC patients (11, 12). Moreover, fibrosis in tumor tissue may lead to exclusion of cytotoxic immune cells from accessing the cancer cells and killing them. This mechanism of immune exclusion is one of the leading causes of immunotherapy resistance (23). Therefore, we aimed to assess the hypothesis that COMP expression in CRC leads to fibrosis-driven exclusion of tumor infiltrating immune cells.

In the current study, we aimed to examine the potential association between elevated levels of COMP in tumors from patients with CRC and levels of fibrosis, tumor-infiltrating populations of immune cells, and expression of PD-L1 on immune cells and tumor cells. The results revealed that COMP expression was an independent prognostic factor for OS. Moreover, COMP expression was associated with fewer tumor-infiltrating immune cells and was negatively correlated with the expression of PD-L1. Tumors with high levels of COMP expression had more fibrotic tissue, which appears to prevent the infiltration of the immune cells.

## Materials and methods

### Patient cohort

The study cohort includes all cases of incident CRC in the Malmö Diet and Cancer Study up until 2008 (n=626). Tumor tissue microarrays (TMA) were constructed from 557 cases as previously described (24–28). The primary aim of the Malmö Diet and Cancer Study (MDCS) was to examine relationships between dietary factors and cancer occurrence. The MDCS cohort included non-participants in the European Prospective Investigation into Cancer (EPIC) cohort and is a prospective, population-based cohort. The studied group consisted of 30446 participants, from which 18326 were women (60.2%), and 12120 were men (39.8%). The follow-up of the patients was from 1991 up until 31 December 2008. The information on CRC incidence was acquired from the Swedish Cancer Registry until 31 December 2007, and from The Southern Swedish Regional Tumor Registry from 1 January to 31 December 2008. Patient medical records were used as a source of clinical and treatment data while pathology records served as a source of histopathological data. All tumors with available slides and/or paraffin blocks were re-evaluated histopathologically using haematoxylin and eosin-stained slides. TNM staging followed the guidelines of the American Joint Committee on Cancer. The right colon was defined as the appendix, caecum, and ascending and 2/3 of transverse colon, whereas the left colon was defined as the left colic flexure and, descending and sigmoid colon. The median age at diagnosis was 71 (range 50–86) years. Information on vital status and cause of death was obtained from the Swedish Cause of Death Registry up until December 31, 2013. Follow-up began at CRC diagnosis and ended at death, emigration, or December 31, 2013, whichever came first. Median follow-up time was 5.97 (range 0–21.69) years for the whole cohort (n=5626) and 10.05 (range 5.03–21.69) years for patients remaining alive (n=5274). This population-based cohort has inherent risk of selection bias compared with the general population. The cancer incidence was lower in the participants of the study comparing with the non-participants. Further, mortality was higher in the general population during and after the enrolment period. These concerns were previously addressed and described (24, 25, 27). The Ethics committee of Lund University (ref nr 51/90 and 530/08) approved the study.

### Immunohistochemical staining

**Figure 1:**
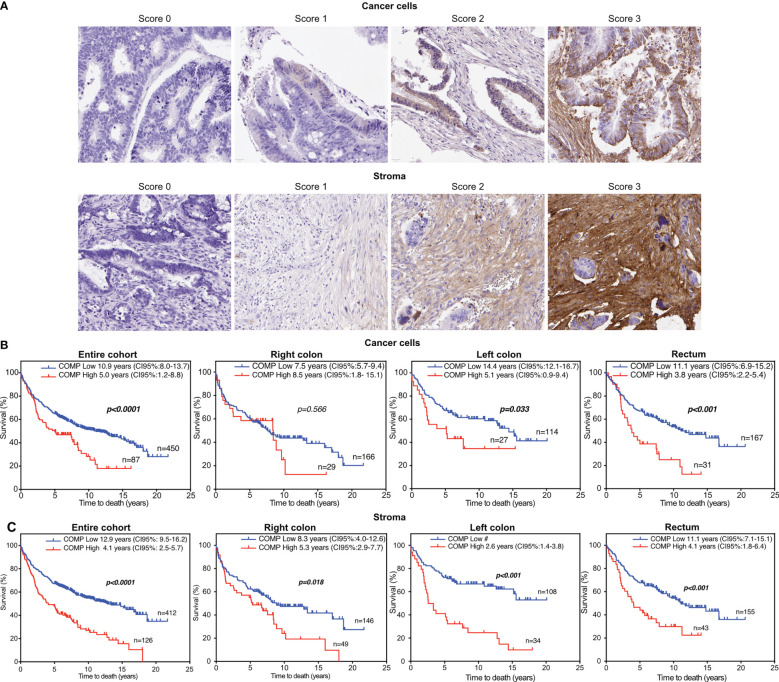
The overall survival (OS) of colorectal cancer patients was reduced when high levels of COMP expression were detected in tumors using immunohistochemistry. **(A)** Representative images of the tumor samples stained for COMP expression. The staining intensity was evaluated separately in the cancer cells and in stroma. For the estimation of OS, the Kaplan-Meier analysis was used to compare low (score 0-1) to high (score 2-3) expression of COMP by the cancer cells **(B)** or in stroma **(C)**. Similar correlation was observed when OS was estimated considering the anatomical site of the primary tumor. Bold indicates significant p-values lower than 0.05 and 95% confidence intervals are provided in brackets. # Incalculable.

TMA blocks were sliced and placed on slides followed by antigen retrieval with citric acid buffer at pH 6. All slides were stained simultaneously using the same aliquot of diluted anti-COMP rabbit polyclonal antibody purified using affinity-chromatography with immobilized antigen and previously characterized for its high specificity (5). The Aperio Scanner (Leica) at 40X was used to scan stained TMAs. The intensity of COMP staining was evaluated independently and blindly by two observers. Staining in cancer cells was scored separately from staining in stroma since we previously observed that these may have different correlations with clinical parameters depending on the type of cancer studied. The score scale ranged from 0 (no expression) to 3 (highest expression; **Figure 1A**). Furthermore, TMAs staining were also evaluated for COMP expression with the QuPath software (29). The total (cancer cells and stroma) percentage of positive cells per TMA core was used as a quantitative unbiased measurement. The patient’s samples were dichotomized as low COMP expressing and high COMP expressing according to the median.

Tumor-infiltrating immune cells and PD-L1 expression on both immune and tumor cells were characterized in previous studies (24, 25, 28). The antibodies used in these staining were: anti-PD-L1 (clone E1L3N, dilution 1:200, Cell Signaling Technologies), anti-CD8 (clone C8/144B, dilution 1:50, Dako), anti-FoxP3 (clone236A/E7, dilution 1:200, Abcam), anti-CD3 (clone 2GV6, prediluted, Ventana Medical Systems), anti-CD20cy (Clone L6, prediluted, Ventana Medical Systems), anti-CD138 (clone MI15, prediluted, Ventana Medical Systems), anti-CD56 (Clone MRQ-42, pre-diluted, Ventana Medical Systems), anti-CD163 (clone 10D6, diluted 1:200, Novus Biologicals), and CD68 (clone KP1, diluted 1:1000, Dako). Previous studies were also source of information on MSI instability screening status assessed by immunohistochemistry (26) and *KRAS* and *BRAF* mutation status determined by pyrosequencing (30).

### Collagen staining

A sequential slice of the TMAs was stained for collagen expression. In brief, 0.1% Sirius Red (Sigma-Aldrich) was used to stain the collagen fibers red. The non-collagenous proteins were stained green with 0.04% Fast Green (Merck), and Weigert’s hematoxylin nuclear staining solution (Histolab) was used to detect cell nuclei. The intensity of collagen staining, and fiber organization were evaluated by FIJI macro TWOMBLI combined with QuPath open software TMA images processing (31).

### Statistical analyses

Non-parametric tests were used for the statistical evaluation of the data. When two groups were compared, the Mann-Whitney test was used. For comparison of more than two groups, the Kruskall-Wallis test was used. Correlations between two variables were evaluated with the Spearman’s rank correlation. The Kaplan-Meier plot and log rank analysis was used for estimation of survival when one variable was evaluated while the Cox regression proportional hazard models were used to estimate the hazard ratios for death according to COMP expression adjusted for multiple variables. The calculations were performed with IBM SPSS Statistics for Mac, Version 28.

## Results

### COMP expression in colorectal tumors is associated with the grade and the stage of the disease

Of a total 626 samples, COMP staining of cancer cells could be evaluated in 536 samples and in the stroma in 537 samples, due to tissue detachment distributed randomly between the slides, or because cancer cells or stroma were not present in some samples (2 samples). COMP was expressed (score 1-3) in the cancer cells in 39.5% of the patients and in the stroma in 60% of the patients (**Figure 1A**). COMP expression was not correlated with anatomical origin. Higher levels of COMP expression by the cancer cells and in the stroma were associated with the stage of the primary tumor (T-stage, p=0.001 for cancer cells, and p=0.002 for the stroma), regional lymph node metastasis (N-stage p=0.002 for cancer cells and p<0.001 for the stroma), and the presence of distant metastasis (M-stage, p=0.002 for cancer cells and p= 0.007 for the stroma; **Table 1**). Moreover, the expression of COMP was correlated with a higher tumor grade irrespectively of the source of expression (p=0.045 for cancer cells and p=0.027 for the stroma; **Table 1**). The presence of COMP in the tumor stroma correlated with MSI (p=0.024). COMP expression in cancer cells or stroma was not associated with *KRAS* or *BRAF* mutations.

**Table 1:** COMP expression in the entire cohort and correlations with the clinical characteristics and associations of the patients.

	Cancer cells										Stroma								
_Factor_^Score^	0		1		2			3		*p*-value	0		1		2		3		*p*-value
All (N=537)	N	%	N	%		N	%	N	%		N	%	N	%	N	%	N	%	
Age at diagnosis										0.662^b^									0.448^b^
<50	1	0.2	0	0.0		0	0.0	0	0.0		1	0.2	0	0.0	0	0.0	0	0.0	
50-70	150	28.0	60	11.2		26	4.9	10	1.9		93	17.3	99	18.4	39	7.3	17	3.2	
>70	173	32.3	65	12.1		39	7.3	12	2.2		121	22.5	97	18.1	55	10.2	15	2.8	
Sex										0.135^a^									0.105^a^
Female	177	33.0	65	12.1		30	5.6	9	1.7		121	22.5	101	18.8	46	8.6	14	2.6	
Male	147	27.4	60	11.2		35	6.5	13	2.4		94	17.5	95	17.7	48	8.9	18	3.4	
Location										0.998^b^									0.817^b^
Right colon	116	21.7	50	9.4		24	4.5	5	0.9		84	15.7	62	11.6	40	7.5	9	1.7	
Left colon	87	16.3	27	5.1		21	3.9	6	1.1		54	10.1	54	10.1	27	5.0	7	1.3	
Rectum	119	22.3	48	9.0		20	3.7	11	2.1		75	14.0	80	15.0	27	5.0	16	3.0	
T-stage										0.001^b^									0.002^b^
1	37	7.1	11	2.1		0	0.0	0	0.0		27	5.2	17	3.3	4	0.8	0	0.0	
2	46	8.9	11	2.1		4	0.8	2	0.4		28	5.4	26	5.0	8	1.5	1	0.2	
3	193	37.2	73	14.1		51	9.8	13	2.5		128	24.6	118	22.7	61	11.7	25	4.8	
4	40	7.7	23	4.4		9	1.7	6	1.2		23	4.4	30	5.8	18	3.5	6	1.2	
N-stage										0.002^b^									<0.001^b^
0	189	38.3	64	13.0		30	6.1	6	1.2		130	26.3	113	22.8	36	7.3	10	2.0	
1	69	14.0	25	5.1		15	3.0	12	2.4		33	6.7	46	9.3	26	5.3	17	3.4	
2	40	8.1	22	4.5		18	3.6	4	0.8		26	5.3	27	5.5	26	5.3	5	1.0	
M-stage										0.002^a^									0.007^a^
0	278	52.6	99	18.7		46	8.7	18	3.4		184	34.7	165	31.1	71	13.4	23	4.3	
1	40	7.6	26	4.9		18	3.4	4	0.8		27	5.1	29	5.5	22	4.2	9	1.7	
Vascular invasion										0.187^a^									0.017^a^
No	91	29.1	35	11.2		20	6.4	4	1.3		58	18.5	63	20.1	25	8.0	5	1.6	
Yes	89	28.4	40	12.8		21	6.7	13	4.2		48	15.3	66	21.0	31	9.9	18	5.7	
KRAS										0.162^a^									0.188^a^
Wild type	199	39.3	79	15.6		33	6.5	10	2.0		133	26.3	120	23.7	51	10.1	17	3.4	
Mutated	107	21.1	39	7.7		30	5.9	9	1.8		70	13.8	64	12.6	38	7.5	13	2.6	
BRAF										0.680^a^									0.421^a^
Wild type	259	51.3	99	19.6		54	10.7	18	3.6		169	33.5	159	31.5	77	15.2	26	5.1	
Mutated	46	9.1	19	3.8		9	1.8	1	0.2		33	6.5	25	5.0	12	2.4	4	0.8	
Differentiation										0.045^a^									0.027^a^
Low grade	62	11.8	27	5.1		21	4.0	6	1.1		39	7.4	41	7.8	26	4.9	11	2.1	
High grade	256	48.6	95	18.0		44	8.3	16	3.0		170	32.2	152	28.8	68	12.9	21	4.0	
Microsatellite stability										0.135^a^									0.024^a^
Stable	253	50.4	101	20.1		54	10.8	20	4.0		165	32.8	156	31.0	79	15.7	28	5.6	
Unstable	49	9.8	18	3.6		7	1.4	0	0.0		38	7.6	25	5.0	11	2.2	1	0.2	

COMP, cartilage oligomeric matrix protein; The bold indicates p-values <0.05, ^a^Calculated with Mann–Whitney U two-tailed exact p-value. ^b^Calculated with Kruskal-Wallis exact p-value.

Analyses were also performed according to anatomical tumor origin. Expression of COMP by cancer cells was denoted in 40.5% of patients with tumors in the right colon, in 38.3% of patients with tumors in the left colon, and in 39.9% of patients with tumors in the rectum. Expression of COMP in the stroma was denoted in 57% of patients with tumors in the right colon, in 62.0% of patients with tumors in the left colon, and in 62.1% of patients with tumors in the rectum. As shown in **Table S1**, correlations between COMP expression both in tumor cells and in the stroma and clinical characteristics, were mainly seen in tumors of the right colon (**Tables S1–3**).

### Patients with high levels of COMP expression had shorter survival irrespectively of the anatomical site of the primary tumor

**Figure 2:**
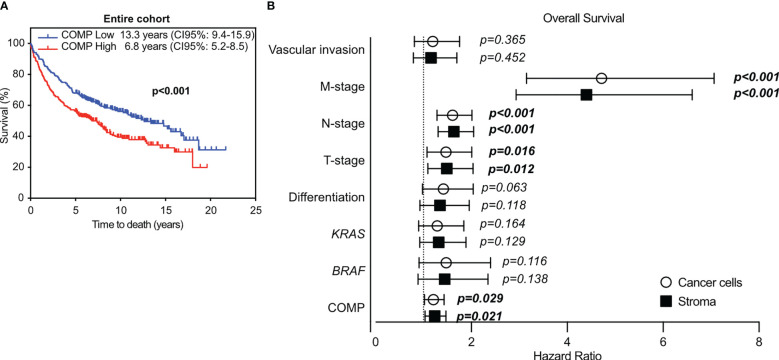
The immunostaining for COMP was evaluated with QuPath software. The expression of COMP was quantified as the percentage of total positive cells (cancer cells and stroma) per TMA core. Based on the calculated median, the tumor samples were stratified into low or high COMP expression groups. **(A)** Patients’ survival was evaluated with the Kaplan-Meier estimator, with the log-rank test. **(B)** Cox multivariable COMP analyses in relation to disease progression markers in the entire cohort, utilizing the score system. The bold indicates *p*-values <0.05.

For survival analyses, patients were stratified into groups with low (score 0-1) and high (score 2-3) COMP expression. Kaplan-Meier analyses revealed that patients with high tumor-cell specific COMP expression had a shorter median OS of 5 years compared with 10.9 years for patients with low tumor-cell specific COMP expression (p<0.0001; **Figure 1B**). Similarly, patients with high levels of COMP expression in the stroma had a median OS of 4.1 years compared to the 12.9 years median OS for patients with low levels of COMP expression (p<0.0001; **Figure 1C**). Similar association of COMP expression with shorter OS of patients was observed when patients were stratified by the anatomical site of the primary tumor (the right and the left colon tumors, **Figure 1B**). The only exception were patients with a primary tumor in the right colon, for whom the expression of high levels of COMP in cancer cells appeared not to influence the OS. Furthermore, we used unbiased quantitative evaluation of COMP expression with the QuPath software calculating the percentage of total (cancer cells and stroma) positive cells. The survival analyses with the Kaplan-Meier consistently revealed that patients with high percentage of COMP positive cells in tumors had shorter median OS (p<0.001) in comparison with patients with low percentage of COMP positive cells (**Figure 2A**).

In multivariable Cox proportional hazards analyses, COMP expression in the cancer cells (p=0.029) and in the stroma (p=0.021) was associated with reduced patient OS with a hazard ratio of 1.2, after adjustment for *BRAF* and *KRAS* mutations, tumor differentiation, TNM-stage, and vascular invasion (**Figure 2B**). When the anatomical site of the primary tumors was considered in multivariable Cox proportional hazards analyses, the expression of COMP was not prognostic in patients with primary right colon tumors (**Table S4**). In patients with primary left colon tumors, the expression of COMP by the cancer cells remained prognostic (p=0.002). COMP was prognostic of OS in patients with rectum primary tumors when COMP was expressed in the stroma (p=0.014).

### Less immune cells are infiltrating the tumor microenvironment in COMP-rich cancer cells and stroma

**Figure 3:**
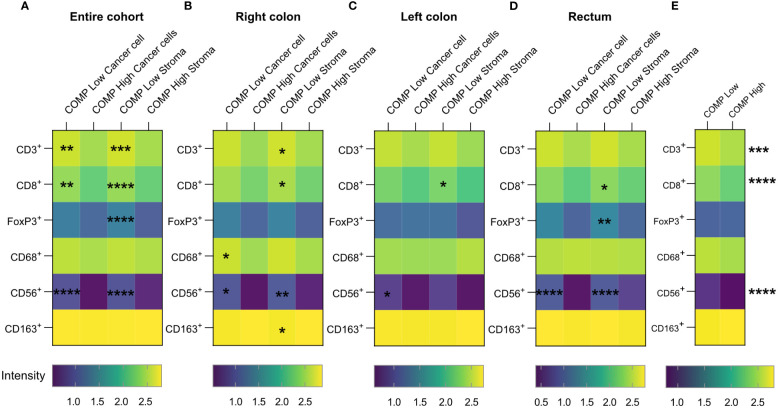
Numbers of infiltrating immune cells decrease in colorectal cancer tumors expressing COMP. **(A)** Heatmaps correlating the infiltrating population of immune cells with the levels of COMP expression by the cancer cells or in stroma. **(B–D)** The same analysis was performed concerning the anatomical site of the primary tumor. Spearman’s regression analysis was used. COMP low: patients with score 0-1; COMP high: patients with score 2-3. **(E)** The Heatmap depicts the correlation of infiltrating immune cell populations with the total (cancer cells and stroma) percentage of COMP positive cells. The immunostaining for COMP was evaluated with QuPath software. COMP low: patients with percentage of positive cells smaller than the median; COMP high: patients with percentage of positive cells higher than the median. The heatmap scale bar represents the estimated log10 of intensity; Intensity represents the total number of infiltrating lymphocytes; *p≤0.05, **p≤0.01, ***p≤0.001 and ****p≤0.0001.

To investigate the effect of COMP expression on immune cell exclusion in CRC, we combined the COMP expression data with already available information about tumor-infiltrating immune cells (28). High levels of COMP expression by the cancer cells and in the stroma were associated with fewer tumor-infiltrating T-cells that were CD3^+^ (p=0.010 cancer cells and p<0.001 stroma), CD8^+^ (p=0.007 cancer cells and p<0.001 stroma) as well as CD56^+^ natural killer cells (NK; p<0.001 cancer cells and p<0.001 stroma) (**Figure 3A**). Additionally, high expression of COMP in the stroma was associated with less infiltrating FoxP3^+^ T-cells (p=0.016, **Figure 3A**). Right colon primary tumors had less CD3^+^ (p=0.042), and CD8^+^ (p=0.016) infiltrating T-cells and CD163^+^ macrophages (p=0.025) when high levels of COMP were found in the stroma (**Figure 3B**). When high levels of COMP were evident in both cancer cells and the stroma, fewer infiltrating CD56^+^ NK cells were detected (p=0.022 cancer cells and p=0.002 stroma). CD68^+^ macrophages also infiltrated less in the presence of high COMP expression by the cancer cells (p=0.033). Left colon primary tumors were infiltrated with fewer CD8^+^ T-cells (p=0.045) when high COMP expression was detected in the stroma (**Figure 3C**). Also, high levels of COMP expression in the cancer cells were associated with fewer CD56^+^ NK cells (p=0.044). Primary tumors in the rectum were characterized by less infiltrating CD8^+^ (p=0.030) and FoxP3^+^ T-cells (p=0.008) when COMP was present in the stroma (**Figure 3D**). CD56^+^ NK cells were infiltrating the rectum tumors less when COMP was highly expressed by the cancer cell and in the stroma (p<0.001 cancer cells and p<0.001 stroma). When the total COMP expression was evaluated using QuPath software, an unbiased quantitative method, a low percentage of COMP expressing cells was correlated with more infiltrating CD3^+^ or CD8^+^ T-cells and CD56^+^ natural killer cells (**Figure 3E**).

### Expression of the PD-L1 is negatively correlated with the expression of COMP

PD-1 is a receptor expressed by T-cells and its stimulation leads to T-cell inactivation. In contrast, its ligand PD-L1 is expressed by cancer and other immune cells. Upregulation of PD-L1 expression by cancer or immune cells has been correlated with negative patient prognosis in colorectal cancer (28). To determine whether reduced infiltration of T-cells in the COMP-positive tumors was correlated with the expression of PD-L1, we analyzed previously collected data (28). Interestingly, PD-L1-expressing immune cells were negatively correlated with the expression of COMP by the cancer cells and in the stroma (p<0.001; **Table 2**). A similar negative association was evident when we compared the PD-L1-expressing cancer cells with the expression of COMP in the stroma. CRC patients with rectum primary tumors presented a similar negative association between the expression of PD-L1 by the immune cells and the expression of COMP both by the cancer cells and in the stroma. For patients with right colon primary tumors, we found a negative correlation between the PD-L1-expressing cancer cells and the levels of COMP in the stroma (**Table S5**). These results suggest that the mechanism leading to a decrease in tumor-infiltrating T-cells when high levels of COMP are expressed by the cancer cells and in the stroma was independent of the immunosuppressive effect of PD-L1.

**Table 2:** Evaluation of COMP and PD-L1 associated expression in the whole cohort.

	Cancer cells				*p*-value	Stroma				*p*-value
	COMP Low		COMP High			COMP Low		COMP High		
	N	(%)	N	(%)		N	(%)	N	(%)	
PD-L1 immune cells					<0.001					<0.001
0-9%	175	33.5%	54	10.3%		153	29.3%	76	14.5%	
10-49%	159	30.5%	21	4.0%		146	27.9%	34	6.5%	
50-100%	102	19.5%	11	2.1%		102	19.5%	12	2.3%	
PD-L1 cancer cells					0.351					0.045
<1%	345	66.1%	73	14.0%		314	60.0%	104	19.9%	
1-4%	44	8.4%	6	1.1%		41	7.8%	9	1.7%	
5-9%	11	2.1%	5	1.0%		11	2.1%	6	1.1%	
10-49%	13	2.5%	0	0.0%		13	2.5%	0	0.0%	
50-100%	23	4.4%	2	0.4%		22	4.2%	3	0.6%	

COMP, cartilage oligomeric matrix protein; PD-L1, programmed death-ligand 1; PD-1, Programmed cell death protein. Calculated with Kruskal-Wallis exact p-value. The bold indicates p-values <0.05.

### Prognostic significance of COMP levels after adjustment for PD-L1 expression from the immune cells or cancer cells and other markers of the immune cells

We then aimed to investigate the prognostic value of COMP expression levels in CRC tumors corrected for PD-L1 expression by immune cells and cancer cells, and the different markers of immune cells populations (CD3^+^, CD8^+^, FoxP3^+^, CD68^+^, CD56^+^, and CD163^+^). The elevated levels of COMP expression by the cancer cells (HR 1.190, p=0.0016) and in stroma (HR 1.295, p<0.001) were predictive of worse OS of the CRC patients (**Table 3**). A similar result was determined for patients with primary rectum tumors. Further, COMP expression in the stroma was a predictive marker of OS for patients with primary left colon tumors (**Table S5**).

**Table 3:** Cox multivariable analyses with covariables related to the infiltrating immune cells.

Survival	Cancer cells					Stroma			
Variable	HR	95% CI			*p*-value	HR	95% CI		*p*-value
COMP	1.190	1.033	1.371		**0.016**	1.295	1.121	1.497	**<0.001**
PD-L1 immune cells	0.644	0.524	0.791		**<0.001**	0.685	0.556	0.844	**<0.001**
PD-L1 cancer cells	1.046	0.915	1.197		0.508	1.078	0.942	1.234	0.277
CD3^+^	0.694	0.499	0.963		**0.029**	0.712	0.511	0.991	**0.044**
CD8^+^	1.311	0.941	1.829		0.110	1.307	0.935	1.827	0.117
FoxP3^+^	0.791	0.607	1.030		0.082	0.810	0.622	1.055	0.118
CD68^+^	0.827	0.622	1.099		0.190	0.774	0.581	1.031	0.080
CD56^+^	0.894	0.688	1.162		0.401	0.940	0.720	1.227	0.649
CD163^+^	1.918	1.431	2.569		**<0.001**	1.725	1.281	2.323	**<0.001**

COMP, cartilage oligomeric matrix protein. The bold indicates p-values <0.05.

### Denser fibrosis correlates with the levels of COMP expression and decrease in infiltrating immune cells

**Figure 4:**
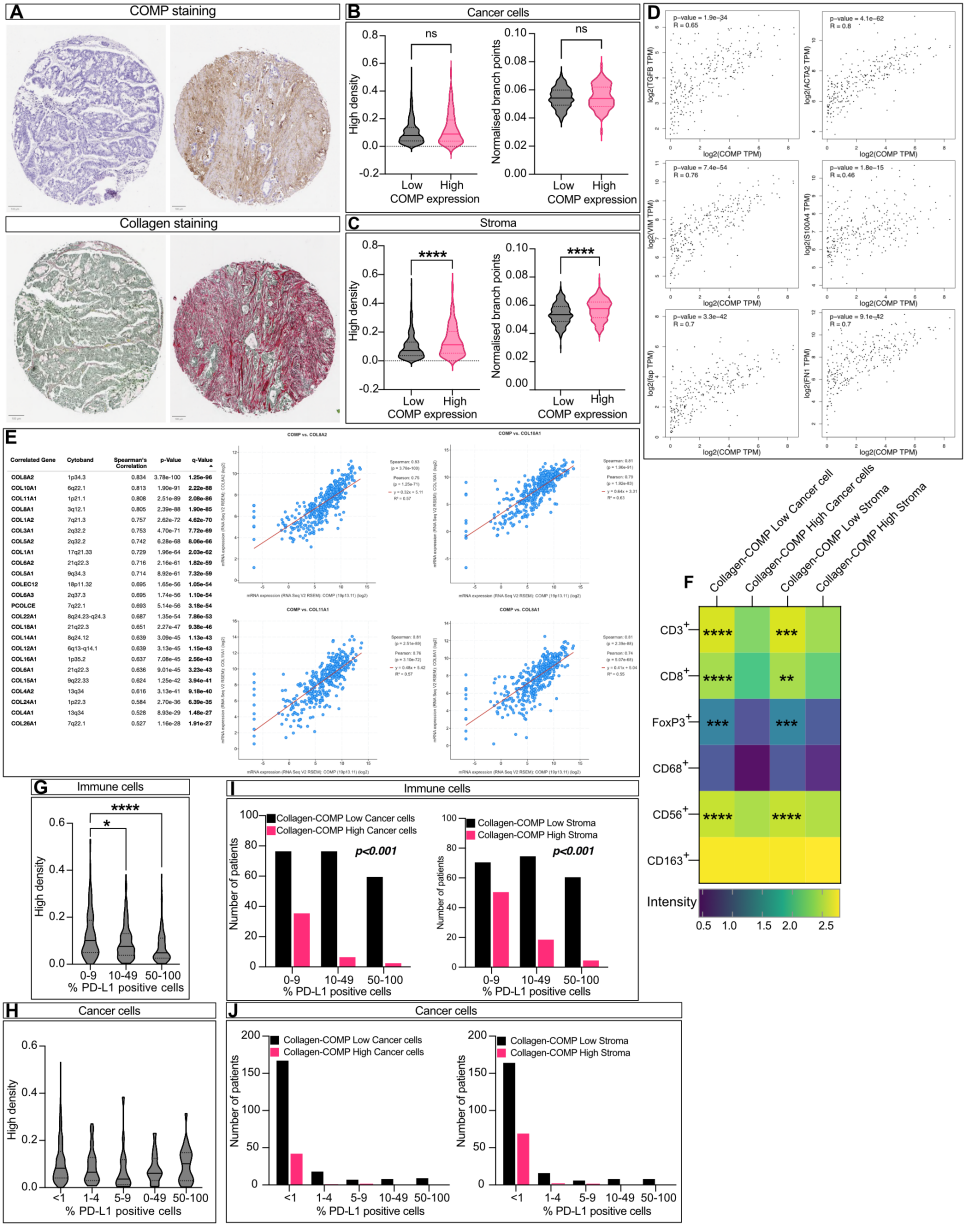
COMP expression correlates with collagen deposits. **(A)** Sequential cuts of TMA slides were stained with Sirius Red detecting collagen and counterstained with Fast Green labelling generally non-collagenous proteins. The density and the organization of collagen fibers was evaluated by macro TWOMBLI in FIJI. Violin plot comparing the fibers density and organization when COMP was expressed by the cancer cells **(B)** (n=265) or in the stroma **(C)** (n=290; Mann-Whitney U test). **(D)** Co-expression analysis was performed with the GEPIA2 online tool between *COMP* RNA expression levels and various fibrosis markers. The p-value was evaluated with Spearman’s correlation. **(E)** The cBioPortal and the TCGA Firehose Legacy study were utilized to analyze the correlation of *COMP* with different collagen gene RNA expressions. **(F)** Tumors with high density of collagen fibres and high levels of COMP expression (Collagen-COMP high) contained fewer immune cells in comparison to tumors with low collagen fibers dense and low COMP levels (Collagen-COMP low; Spearman’s regression analysis). **(G)** Dense collagen fibres were negatively correlated with PD-L1 positive immune cells, **(H)** but were not correlated with the number of positive PD-L1 cancer cells (Kruskal-Wallis test). **(I)** Collagen-COMP high tumors were negatively correlated with the percentage of PD-L1 positive immune cells (Kruskal-Wallis test). **(J)** In contrast, no correlation was observed with the percentage of PD-L1 positive cancer cells (Kruskal-Wallis test). The heatmap scale bar is represents the estimated log10 of intensity; Intensity represents the total number of infiltrating lymphocytes; *p≤0.05, **p≤0.01, ***p≤0.001 and ****p≤0.0001.

Next, we assessed the levels of tissue fibrosis by Sirius red-Fast Green staining (**Figure 4A**) and evaluated the collagen fibers’ organization and density using the TWOMBLI plugin of FIJI. High levels of COMP expression in the stroma, but not in cancer cells, were correlated with collagen fiber density and the number of branch points (**Figures 4B, C**). In addition, we used the GEPIA2 online tool (32) to analyze the RNA sequencing expression data of the TCGA and the GTEx projects. COMP expression was consistently and strongly correlated with the expression of several markers of fibrosis (TGF-β, α-SMA, fibroblast activation protein, S100A4, vimentin, and fibronectin; **Figure 4D**). Furthermore, COMP expression was positively correlated with 23 different types of collagens when their co-expression was investigated with the cBioPortal (**Figure 4E**) analyzing RNA expression data from the TCGA project stratified for colorectal cancer patients (TCGA, Firehose Legacy). To evaluate the combined effect of COMP and tumor fibrosis on the infiltrating immune cells, we stratified the patients into four groups: 1) low collagen density and low COMP expression in stroma (collagen-COMP low stroma), 2) high collagen density and high COMP expression in stroma (collagen-COMP high stroma), 3) low collagen density and low COMP expression from cells (collagen-COMP low cells), 4) high collagen density and high COMP expression in cells (collagen-COMP high cells). Tumors in the collagen-COMP high groups had fewer infiltrating T-cells that were CD3^+^ (p<0.001 for both cancer cells and stroma), CD8^+^ (p<0.001 cancer cells and p=0.002 stroma), and FoxP3^+^ (p<0.001 for both cancer cells and stroma) compared to tumors in the collagen-COMP low groups. Also, fewer infiltrating CD56^+^ natural killer cells (p<0.001; for both cancer cells and stroma) were detected in the tumor samples (**Figure 4F**). These data indicate a potential synergetic effect of COMP expression and tissue fibrosis in excluding immune cells from infiltrating the tumors. Interestingly, tumors with a high density of collagen fibers were less infiltrated by PD-L1 positive immune cells (**Figures 4G, H**). A similar phenomenon was observed in the group of collagen-COMP high tumors compared to collagen-COMP low tumors, irrespective of whether COMP was expressed by tumor cells or in stroma (**Figures 4I, J**).

## Discussion

In recent years, COMP has emerged as a novel regulator of cancer progression in several different types of cancer. Among these was CRC, for which COMP expression was a prognostic marker of decreased OS (11, 13–16). In the current study, we stained primary tumors for COMP expression and collagen density using TMAs derived from a large cohort of CRC patients, allowing us to confirm the association of COMP expression with poor OS. Analyses of COMP expression in relation to the clinicopathological characteristics of the patients revealed that COMP expression both by the cancer cell and in the stroma was associated with TNM- stage and grade of tumor differentiation. The obtained results, correlating COMP expression with shorter OS, and correlating COMP expression with the clinicopathological characteristics of the patients, were in accordance with published observations (11, 13–16). Further, we showed that COMP expression correlates with increased collagen fibrosis and decreased infiltration of several types of immune cells. The latter was not dependent on altered PD-L1 expression, and we thus hypothesize that a COMP-dependent increase in fibrosis may result in immune exclusion.

Our results are in accordance with past observations, but a larger, population-based, prospective patient cohort was analyzed in the current study. Further, previous studies used mainly mRNA expression data to determine COMP expression in tumors as a whole, while we detect COMP separately in tumor cells and stroma using a validated antibody. The underlying molecular mechanism of the oncogenic effect of COMP has been partially revealed in previous studies. COMP overexpression in colon cancer cell lines was linked with the promotion of cell proliferation by altering the PI3K-Akt-mTOR axis (11). Moreover, COMP expression in CRC was associated with the induction of epithelial-mesenchymal transition *in vivo* and *in vitro* (12). In breast cancer, COMP expression was shown to expand the population of cancer stem cells *via* bridging Notch3 with Jagged1, resulting in the activation of the Notch pathway (7).In the cartilage and presumably tumor tissue, COMP binds to collagen fibers, organizes them, and thus makes the cartilage denser and stiffer (4). Recently we revealed that periampullary adenocarcinomas expressing high levels of COMP had denser collagenous matrix correlating with the immune exclusion of T-cells from the cancer cells compartment of the tumors (22). Now, we observed a similar phenomenon in patients with CRC i.e., that CD8^+^ T-cells infiltrated poorly into tumors expressing COMP. PD-1 is a receptor expressed by T-cells and its activation by its ligand, PD-L1, leads to inhibition of cytotoxic activity of activated T-cells. PD-L1 is expressed by several cell types, including cancer cells. Induction of PD-L1 expression by cancer cells correlates with worse survival of cancer patients (33, 34). Moreover, PD-L1 can be expressed by immune cells reducing antitumor activity (35, 36). In order to escape T-cell killing, cancer cells hijack the IFN-γ pathway. NK cells produce IFN-γ to activate major histocompatibility complex expression from the cancer cells and enhance neoantigen presentation. On the other hand, cancer cell stimulation with IFN-γ upregulates the expression of PD-L1, leading to inactivation of cytotoxic T-cells (37). Interestingly, COMP expression was negatively correlated with PD-L1-positive cancer cells and immune cells. Since COMP deposition in the stroma was positively associated with denser collagen deposits, a possible explanation could be that dense collagen prevents T-cell infiltration, independent of PD-L1 expression. This agrees with previous studies showing that fibrosis in peritoneal or lymph node metastases in CRC prevents the infiltration of lymphocytes (38, 39). Also, primary stroma-rich CRC tumors had fewer infiltrating immune cells (40), and preoperative suppression of tumor-specific CD4^+^ T-cells was correlated with patient relapse (41). We must note that we cannot exclude a direct immune suppressive effect of COMP on T-cells, especially considering that COMP prevents the phagocytosis and killing of M. catarrhalis by human neutrophils (42), opening the possibility that COMP regulates the function of various immune cell types. This is supported by the observation that while COMP expression from both cancer cells and in stroma correlated with immune exclusion, only COMP in stroma correlated with collagen density.

Moreover, COMP expression remained a predictive marker of the patient OS even when adjusted for all the assessed markers of the immune cells (CD3^+^, CD8^+^, FoxP3^+^, CD68^+^, CD56^+^, CD163^+^, and PD-L1), indicating that COMP expression may have several oncogenic effects that influence OS. It is already demonstrated in CRC that COMP promotes cell proliferation *via* activation of Akt-pathway (11) and epithelial to mesenchymal transition (12, 15).

An interesting new observation made in this study was that COMP expression in stroma correlates with microsatellite instability. Microsatellite instability results in tumors with an increased generation of neo-antigens, which can be recognized and removed by activated T-cells (43). In the current study, we correlated COMP expression in the tumor stroma with fibrosis, which could prevent T-cells from infiltrating the denser stroma and eradicating the cancer cells expressing the neo-antigens, a phenomenon observed previously in urothelial cancer (23). Moreover, collagen may interact with the LAIR1 receptor on T-cells, leading to their exhaustion (44). Consequently, inhibition of the effect of collagen on the LAIR1 receptor sensitizes the resistant tumors to PD-L1 immune checkpoint inhibitors (44, 45). Further, TGF-β is a well-documented factor that drives anti-PD-L1 resistance in colon cancer (46). Many studies reported TGF-β to be co-expressed with COMP (4, 47, 48), which could lead to immunosuppression even under microsatellite instability. In contrast, COMP expression by cancer cells was not correlated with MSI. This observation may indicate that COMP-mediated alteration of the tumor microenvironment yields fewer infiltrating immune cells, rather than a direct effect of COMP on the cancer cells. It has been previously shown that PD-L1 expression by cancer cells is associated with MSI (28).

A recent study investigating the tumor microenvironment in CRC used RNA sequencing data from the TCGA GEO datasets and the MCP-counter algorithm to calculate the CAFs abundance in the tumor microenvironment (49). Furthermore, gene expression was studied in the CAFs population in search of prognostic and immunotherapeutic biomarkers and identified COMP as a suitable prognostic candidate. Our immunohistochemical staining showed clear expression of COMP in cancer cells but also extensive deposits in the extracellular matrix. However, the source of COMP found in the stroma is currently unknown. It could originate by secretion from either cancer cells or fibroblasts. Fibroblasts were previously shown to express COMP particularly in fibrosis (19). For pancreatic adenocarcinoma it has been reported that less than 10% of the tumor ECM proteins originate from cancer cells, while the remaining was derived from the CAFs. The proteins secreted by CAFs were the core matrisome, mainly collagen, while those secreted by cancer cells had ECM regulatory functions (50). In line with our current findings, COMP expression in CRC was correlated with fewer infiltrating CD8^+^ T-cells in the TCGA and GSE cohorts (49). Further, COMP correlated with increased infiltration of M2 macrophages, which are tissue-resident macrophages with immunosuppressive functions promoting tissue repair and expressing ECM components, including collagen (49). Our data in turn indicate that CRC tumors expressing high levels of COMP were infiltrated with fewer CD68^+^ macrophages, a marker for both M1 and M2 macrophages. This may suggest that CRC tumors were poorly infiltrated by a pro-inflammatory M1 macrophages. Importantly, low levels of COMP expression in CRC correlated with substantial therapeutic benefits and response to immunotherapy (49).

The current study is limited by the nature of the clinical material analyzed. While human samples from naïve primary tumors provide valuable information that will drive future studies *in vitro* and *in vivo*, they also limit the ability to manipulate the experimental parameters and dissect the molecular mechanisms involved. Therefore, future experimental work is anticipated to elucidate how COMP activity restricts the immune response in the tumor microenvironment. Further, the available data did not allow us to study the mechanism by which COMP expression is triggered during cancer development, which will be interesting to address in the future.

In conclusion, we confirmed in a large cohort of CRC patients that expression of COMP in both cancer cells and in stroma correlates with TNM stage and grade of differentiation. Expression of COMP could serve as a prognostic marker of patients OS. Further, COMP expression correlates with exclusion of several types of immune cells including CD8^+^ T-cells and NK cells from the cancer cell compartment, by a mechanism that is potentially independent of PD-L1 expression. This phenomenon may be explained by the presence of dense collagen deposits detected in COMP expressing tumors, which could prevent the immune cells from infiltrating.

## Data availability statement

The original contributions presented in the study are included in the article/**Supplementary Material**, further inquiries can be directed to the corresponding author.

## Ethics statement

The studies involving human participants were reviewed and approved by The Ethics committee of Lund University (ref nr 51/90 and 530/08) approved the study. The patients/participants provided their written informed consent to participate in this study.

## Author contributions

Conceptualization: KP, AB; Methodology and clinical data: KP, AB, CG, CH, KJ; Investigation: KP, AB, CG, CH; Funding acquisition: AB; Supervision: AB, KJ; Writing – original draft: KP, AB; Writing – review & editing: KJ, CG, CH, JB. All authors contributed to the article and approved the submitted version.

## References

[1] Sung H Ferlay J Siegel RL Laversanne M Soerjomataram I Jemal A. Global cancer statistics 2020: GLOBOCAN estimates of incidence and mortality worldwide for 36 cancers in 185 countries. CA Cancer J Clin (2021) 71:209–49. doi: 10.3322/caac.21660 33538338

[2] Guinney J Dienstmann R Wang X de Reynies A Schlicker A Soneson C. The consensus molecular subtypes of colorectal cancer. Nat Med (2015) 21:1350–6. doi: 10.1038/nm.3967 PMC4636487 26457759

[3] Hinshaw DC Shevde LA. The tumor microenvironment innately modulates cancer progression. Cancer Res (2019) 79:4557–66. doi: 10.1158/0008-5472.CAN-18-3962 PMC6744958 31350295

[4] Posey KL Coustry F Hecht JT. Cartilage oligomeric matrix protein: COMPopathies and beyond. Matrix Biol (2018) 71-72:161–73. doi: 10.1016/j.matbio.2018.02.023 PMC6129439 29530484

[5] Englund E Bartoschek M Reitsma B Jacobsson L Escudero-Esparza A Orimo A. Cartilage oligomeric matrix protein contributes to the development and metastasis of breast cancer. Oncogene (2016) 35:5585–96. doi: 10.1038/onc.2016.98 27065333

[6] Papadakos KS Darlix A Jacot W Blom AM. High levels of cartilage oligomeric matrix protein in the serum of breast cancer patients can serve as an independent prognostic marker. Front Oncol (2019) 9:1141. doi: 10.3389/fonc.2019.01141 31737569 PMC6831625

[7] Papadakos KS Bartoschek M Rodriguez C Gialeli C Jin SB Lendahl U. Cartilage oligomeric matrix protein initiates cancer stem cells through activation of Jagged1-Notch3 signaling. Matrix Biol (2019) 81:107–21. doi: 10.1016/j.matbio.2018.11.007 30502484

[8] Englund E Canesin G Papadakos KS Vishnu N Persson E Reitsma B. Cartilage oligomeric matrix protein promotes prostate cancer progression by enhancing invasion and disrupting intracellular calcium homeostasis. Oncotarget (2017) 8:98298–311. doi: 10.18632/oncotarget.21176 PMC5716730 29228690

[9] Li Q Wang C Wang Y Sun L Liu Z Wang L. HSCs-derived COMP drives hepatocellular carcinoma progression by activating MEK/ERK and PI3K/AKT signaling pathways. J Exp Clin Cancer Res (2018) 37:231. doi: 10.1186/s13046-018-0908-y 30231922 PMC6146743

[10] Kuo YH Lai HY Chan TC Hsing CH Huang SK Hsieh KL. Upregulation of cartilage oligomeric matrix protein predicts poor prognosis in urothelial carcinoma. Onco Targets Ther (2022) 15:727–40. doi: 10.2147/OTT.S370028 PMC9252317 35795328

[11] Liu TT Liu XS Zhang M Liu XN Zhu FX Zhu FM. Cartilage oligomeric matrix protein is a prognostic factor and biomarker of colon cancer and promotes cell proliferation by activating the akt pathway. J Cancer Res Clin Oncol (2018) 144:1049–63. doi: 10.1007/s00432-018-2626-4 PMC11813401 29560517

[12] Zhong W Hou H Liu T Su S Xi X Liao Y. Cartilage oligomeric matrix protein promotes epithelial-mesenchymal transition by interacting with transgelin in colorectal cancer. Theranostics (2020) 10:8790–806. doi: 10.7150/thno.44456 PMC7392026 32754278

[13] Nfonsam VN Jecius H Chen D Omesiete PN Ewongwo AN Elquza E. Increasing incidence of colon cancer in the young: assessing the tumor biology. J Am Coll Surg (2019) 229:79–90. doi: 10.1016/j.jamcollsurg.2019.03.022 30995524

[14] Nfonsam VN Jecius HC Janda J Omesiete PN Elquza E Scott AJ. Cartilage oligomeric matrix protein (COMP) promotes cell proliferation in early-onset colon cancer tumorigenesis. Surg Endosc (2020) 34:3992–8. doi: 10.1007/s00464-019-07185-z 31617091

[15] Nfonsam VN Nfonsam LE Chen D Omesiete PN Cruz A Runyan RB. And is associated with poor survival in colon cancer patients. J Surg Res (2019) 233:297–303. doi: 10.1016/j.jss.2018.08.021 30502262

[16] Wusterbarth E Chen Y Jecius H Krall E Runyan RB Pandey R. COMP may be a better prognostic marker than CEACAM5 and correlates with colon cancer molecular subtypes, tumor aggressiveness and overall survival. J Surg Res (2022) 270:169–77. doi: 10.1016/j.jss.2021.09.007 34687957

[17] Barrett T Suzek TO Troup DB Wilhite SE Ngau WC Ledoux P. Mining millions of expression profiles–database and tools. Nucleic Acids Res (2005) 33:D562–6. doi: 10.1093/nar/gki022 PMC539976 15608262

[18] Martinez FJ Collard HR Pardo A Raghu G Richeldi L Selman M. Idiopathic pulmonary fibrosis. Nat Rev Dis Primers (2017) 3:17074. doi: 10.1038/nrdp.2017.74 29052582

[19] Farina G Lemaire R Korn JH Widom RL. Cartilage oligomeric matrix protein is overexpressed by scleroderma dermal fibroblasts. Matrix Biol (2006) 25:213–22. doi: 10.1016/j.matbio.2006.01.007 16520029

[20] Vuga LJ Milosevic J Pandit K Ben-Yehudah A Chu Y Richards T. Cartilage oligomeric matrix protein in idiopathic pulmonary fibrosis. PloS One (2013) 8:e83120. doi: 10.1371/journal.pone.0083120 24376648 PMC3869779

[21] Schulz JN Nuchel J Niehoff A Bloch W Schonborn K Hayashi S. COMP-assisted collagen secretion–a novel intracellular function required for fibrosis. J Cell Sci (2016) 129:706–16. doi: 10.1242/jcs.180216 26746240

[22] Papadakos KS Lundgren S Gialeli C Micke P Mezheyeuski A Elebro J. Expression of cartilage oligomeric matrix protein in periampullary adenocarcinoma is associated with pancreatobiliary-type morphology, higher levels of fibrosis and immune cell exclusion. Oncoimmunology (2022) 11:2111906. doi: 10.1080/2162402X.2022.2111906 35990519 PMC9389925

[23] Mariathasan S Turley SJ Nickles D Castiglioni A Yuen K Wang Y. TGF-beta attenuates tumour response to PD-L1 blockade by contributing to exclusion of T cells. . Nat (2018) 554:544–8. doi: 10.1038/nature25501 PMC6028240 29443960

[24] Berntsson J Svensson MC Leandersson K Nodin B Micke P Larsson AH. The clinical impact of tumour-infiltrating lymphocytes in colorectal cancer differs by anatomical subsite: a cohort study. Int J Cancer (2017) 141:1654–66. doi: 10.1002/ijc.30869 PMC5601279 28677162

[25] Berntsson J Nodin B Eberhard J Micke P Jirstrom K. Prognostic impact of tumour-infiltrating b cells and plasma cells in colorectal cancer. Int J Cancer (2016) 139:1129–39. doi: 10.1002/ijc.30138 27074317

[26] Eberhard J Gaber A Wangefjord S Nodin B Uhlen M Ericson Lindquist K. A cohort study of the prognostic and treatment predictive value of SATB2 expression in colorectal cancer. Br J Cancer (2012) 106:931–8. doi: 10.1038/bjc.2012.34 PMC3305956 22333599

[27] Larsson A Johansson ME Wangefjord S Gaber A Nodin B Kucharzewska P. Overexpression of podocalyxin-like protein is an independent factor of poor prognosis in colorectal cancer. Br J Cancer (2011) 105:666–72. doi: 10.1038/bjc.2011.295 PMC3188928 21829192

[28] Berntsson J Eberhard J Nodin B Leandersson K Larsson AH Jirstrom K. Expression of programmed cell death protein 1 (PD-1) and its ligand PD-L1 in colorectal cancer: relationship with sidedness and prognosis. Oncoimmunology (2018) 7:e1465165. doi: 10.1080/2162402X.2018.1465165 30221062 PMC6136864

[29] Bankhead P Loughrey MB Fernandez JA Dombrowski Y McArt DG Dunne PD. QuPath: open source software for digital pathology image analysis. Sci Rep (2017) 7:16878. doi: 10.1038/s41598-017-17204-5 29203879 PMC5715110

[30] Wangefjord S Sundstrom M Zendehrokh N Lindquist KE Nodin B Jirstrom K. Sex differences in the prognostic significance of KRAS codons 12 and 13, and BRAF mutations in colorectal cancer: a cohort study. Biol Sex Differ (2013) 4:17. doi: 10.1186/2042-6410-4-17 24020794 PMC3846575

[31] Wershof E Park D Barry DJ Jenkins RP Rullan A Wilkins A. A FIJI macro for quantifying pattern in extracellular matrix. Life Sci Alliance (2021) 4. doi: 10.26508/lsa.202000880 PMC7898596 33504622

[32] Tang Z Kang B Li C Chen T Zhang Z. GEPIA2: an enhanced web server for large-scale expression profiling and interactive analysis. Nucleic Acids Res (2019) 47:W556–60. doi: 10.1093/nar/gkz430 PMC6602440 31114875

[33] Ohaegbulam KC Assal A Lazar-Molnar E Yao Y Zang X. Human cancer immunotherapy with antibodies to the PD-1 and PD-L1 pathway. Trends Mol Med (2015) 21:24–33. doi: 10.1016/j.molmed.2014.10.009 25440090 PMC4282825

[34] Thompson RH Gillett MD Cheville JC Lohse CM Dong H Webster WS. Costimulatory B7-H1 in renal cell carcinoma patients: indicator of tumor aggressiveness and potential therapeutic target. Proc Natl Acad Sci USA (2004) 101:17174–9. doi: 10.1073/pnas.040635110 PMC534606 15569934

[35] Cha JH Chan LC Li CW Hsu JL Hung MC. Mechanisms controlling PD-L1 expression in cancer. Mol Cell (2019) 76:359–70. doi: 10.1016/j.molcel.2019.09.030 PMC6981282 31668929

[36] Curiel TJ Wei S Dong H Alvarez X Cheng P Mottram P. Blockade of B7-H1 improves myeloid dendritic cell-mediated antitumor immunity. Nat Med (2003) 9:562–7. doi: 10.1038/nm863 12704383

[37] Garcia-Diaz A Shin DS Moreno BH Saco J Escuin-Ordinas H Rodriguez GA. Interferon receptor signaling pathways regulating PD-L1 and PD-L2 expression. Cell Rep (2017) 19:1189–201. doi: 10.1016/j.celrep.2017.04.031 PMC6420824 28494868

[38] Ikuta D Miyake T Shimizu T Sonoda H Mukaisho KI Tokuda A. Fibrosis in metastatic lymph nodes is clinically correlated to poor prognosis in colorectal cancer. Oncotarget (2018) 9:29574–86. doi: 10.18632/oncotarget.25636 PMC6049853 30038705

[39] Wang E Shibutani M Nagahara H Fukuoka T Iseki Y Okazaki Y. Abundant intratumoral fibrosis prevents lymphocyte infiltration into peritoneal metastases of colorectal cancer. PloS One (2021) 16:e0255049. doi: 10.1371/journal.pone.0255049 34293030 PMC8297902

[40] Ravensbergen CJ Polack M Roelands J Crobach S Putter H Gelderblom H. Combined assessment of the tumor-stroma ratio and tumor immune cell infiltrate for immune checkpoint inhibitor therapy response prediction in colon cancer. Cells (2021) 10(11):2935. doi: 10.3390/cells10112935 34831157 PMC8616493

[41] Betts G Jones E Junaid S El-Shanawany T Scurr M Mizen P. Suppression of tumour-specific CD4(+) T cells by regulatory T cells is associated with progression of human colorectal cancer. Gut (2012) 61:1163–71. doi: 10.1136/gutjnl-2011-300970 PMC3388728 22207629

[42] Liu G Gradstedt H Ermert D Englund E Singh B Su YC. Moraxella catarrhalis evades host innate immunity *via* targeting cartilage oligomeric matrix protein. J Immunol (2016) 196:1249–58. doi: 10.4049/jimmunol.1502071 26712944

[43] Kloor M von Knebel Doeberitz M. The immune biology of microsatellite-unstable cancer. Trends Cancer (2016) 2:121–33. doi: 10.1016/j.trecan.2016.02.004 28741532

[44] Peng DH Rodriguez BL Diao L Chen L Wang J Byers LA. Collagen promotes anti-PD-1/PD-L1 resistance in cancer through LAIR1-dependent CD8(+) T cell exhaustion. Nat Commun (2020) 11:4520. doi: 10.1038/s41467-020-18298-8 32908154 PMC7481212

[45] Horn LA Chariou PL Gameiro SR Qin H Iida M Fousek K. Remodeling the tumor microenvironment *via* blockade of LAIR-1 and TGF-beta signaling enables PD-L1-mediated tumor eradication. J Clin Invest (2022) 132. doi: 10.1172/JCI155148 PMC9012291 35230974

[46] Tauriello DVF Palomo-Ponce S Stork D Berenguer-Llergo A Badia-Ramentol J Iglesias M. TGF-beta drives immune evasion in genetically reconstituted colon cancer metastasis. Nature (2018) 554:538–43. doi: 10.1038/nature25492 29443964

[47] Haudenschild DR Hong E Yik JH Chromy B Morgelin M Snow KD. Enhanced activity of transforming growth factor beta1 (TGF-beta1) bound to cartilage oligomeric matrix protein. J Biol Chem (2011) 286:43250–8. doi: 10.1074/jbc.M111.234716 PMC3234822 21940632

[48] Tran V Karsai A Fong MC Cai W Fraley JG Yik JHN. Direct visualization of the binding of transforming growth factor beta 1 with cartilage oligomeric matrix protein *via* high-resolution atomic force microscopy. J Phys Chem B (2020) 124:9497–504. doi: 10.1021/acs.jpcb.0c07286 PMC7891936 33052673

[49] Ma H Qiu Q Tan D Chen Q Liu Y Chen B. The cancer-associated fibroblasts-related gene COMP is a novel predictor for prognosis and immunotherapy efficacy and is correlated with M2 macrophage infiltration in colon cancer. Biomolecules (2022) 13. doi: 10.3390/biom13010062 PMC9856124 36671447

[50] Tian C Clauser KR Ohlund D Rickelt S Huang Y Gupta M. Proteomic analyses of ECM during pancreatic ductal adenocarcinoma progression reveal different contributions by tumor and stromal cells. Proc Natl Acad Sci USA (2019) 116:19609–18. doi: 10.1073/pnas.1908626116 PMC6765243 31484774

