# Supplementary material for: Expression of Cartilage Oligomeric Matrix Protein in colorectal cancer is an adverse prognostic factor and correlates negatively with infiltrating immune cells and PD-L1 expression
Source: Front Immunol. 2023 May 3;14:1167659. doi: 10.3389/fimmu.2023.1167659 (PMC10188999; doi:10.3389/fimmu.2023.1167659)
Supplement: Supplementary file 1 [file DataSheet_1.pdf]

Table S1. COMP expression in the right colon and correlations with the clinical characteristics and associations of the patients.

	Cancer cells									Stroma								
Factor / Score	0		1		2		3		***p*-value**	0		1		2		3		***p*-value**
All (N=195)	N	%	N	%	N	%	N	%		N	%	N	%	N	%	N	%	
Age at diagnosis									0.193[b]									0.515[b]
<50	0	0.0	0	0.0	0	0.0	0	0.0		0	0.0	0	0.0	0	0.0	0	0.0	
50-70	46	23.6	17	8.7	8	4.1	0	0.0		28	14.4	26	13.3	14	7.2	4	2.1	
>70	70	35.9	33	16.9	16	8.2	5	2.6		56	28.7	36	18.5	26	13.3	5	2.6	
Sex									0.432[a]									0.682[a]
Female	69	35.4	24	12.3	14	7.2	3	1.5		50	25.6	32	16.4	22	11.3	6	3.1	
Male	47	24.1	26	13.3	10	5.1	2	1.0		34	17.4	30	15.4	18	9.2	3	1.5	
T-stage									0.072[b]									0.138[b]
1	7	3.6	2	1.0	0	0.0	0	0.0		6	3.1	2	1.0	1	0.5	0	0.0	
2	12	6.2	6	3.1	0	0.0	0	0.0		9	4.7	8	4.1	1	0.5	0	0.0	
3	76	39.4	25	13.0	18	9.3	2	1.0		54	28.0	35	18.1	26	13.5	7	3.6	
4	20	10.4	16	8.3	6	3.1	3	1.6		14	7.3	17	8.8	11	5.7	2	1.0	
N-stage									**<0.001[b]**									**<0.001[b]**
0	68	36.4	25	13.4	6	3.2	1	0.5		55	29.4	31	16.6	12	6.4	2	1.1	
1	28	15.0	6	3.2	7	3.7	3	1.6		12	6.4	16	8.6	11	5.9	6	3.2	
2	15	8.0	16	8.6	11	5.9	1	0.5		11	5.9	14	7.5	16	8.6	1	0.5	
M-stage									**0.007[a]**									**0.044[a]**
0	99	51.3	36	18.7	16	8.3	3	1.6		73	37.8	46	23.8	31	16.1	5	2.6	
1	16	8.3	14	7.3	7	3.6	2	1.0		11	5.7	15	7.8	8	4.1	4	2.1	
Vascular invasion									0.297[a]									**0.030[a]**
No	32	26.4	16	13.2	3	2.5	1	0.8		24	19.7	21	17.2	8	6.6	0	0.0	
Yes	38	31.4	19	15.7	8	6.6	4	3.3		21	17.2	28	23.0	14	11.5	6	4.9	
KRAS									0.875[a]									0.296[a]
Wild type	67	37.0	32	17.7	13	7.2	2	1.1		51	28.3	37	20.6	20	11.1	5	2.8	
Mutated	40	22.1	15	8.3	10	5.5	2	1.1		27	15.0	19	10.6	17	9.4	4	2.2	
BRAF									0.908[a]									0.719[a]
Wild type	68	37.6	29	16.0	15	8.3	3	1.7		49	27.2	35	19.4	26	14.4	5	2.8	
Mutated	39	21.5	18	9.9	8	4.4	1	0.6		29	16.1	21	11.7	11	6.1	4	2.2	
Differentiation									0.569[a]									0.253[a]
Low grade	40	20.8	14	7.3	11	5.7	3	1.6		26	13.5	22	11.5	15	7.8	5	2.6	
High grade	74	38.5	35	18.2	13	6.8	2	1.0		56	29.2	39	20.3	25	13.0	4	2.1	
Microsatellite stability									0.117[a]									**0.023[a]**
Stable	68	36.6	31	16.7	15	8.1	5	2.7		46	24.7	37	19.9	27	14.5	8	4.3	
Unstable	45	24.2	16	8.6	6	3.2	0	0.0		36	19.4	21	11.3	10	5.4	1	0.5	

Abbreviations: COMP, cartilage oligomeric matrix protein; The bold indicates *p*-values <0.05, [a] Calculated with Mann–Whitney U two-tailed exact *p*-value. [b] Calculated with Kruskal-Wallis exact *p*-value.

Table S2. COMP expression in the left colon and correlations with the clinical characteristics and associations of the patients.

Factor \ Score	Cancer cells 0		1		2		3		*p*-value	Stroma 0		1		2		3		*p*-value
All (N=142)	N	%	N	%	N	%	N	%		N	%	N	%	N	%	N	%	
Age at diagnosis									0.111[b]									0.062[b]
<50	0	0.0	0	0.0	0	0.0	0	0.0		0	0.0	0	0.0	0	0.0	0	0.0	
50-70	47	33.3	14	9.9	7	5.0	2	1.4		30	21.1	30	21.1	9	6.3	2	1.4	
>70	40	28.4	13	9.2	14	9.9	4	2.8		24	16.9	24	16.9	18	12.7	5	3.5	
Sex									0.069[a]									0.273[a]
Female	51	36.2	12	8.5	8	5.7	3	2.1		30	21.1	31	21.8	11	7.7	3	2.1	
Male	36	25.5	15	10.6	13	9.2	3	2.1		24	16.9	23	16.2	16	11.3	4	2.8	
T-stage									**0.002[b]**									0.228[b]
1	20	14.4	2	1.4	0	0.0	0	0.0		11	7.9	10	7.1	1	0.7	0	0.0	
2	5	3.6	0	0.0	2	1.4	1	0.7		3	2.1	3	2.1	2	1.4	0	0.0	
3	47	33.8	20	14.4	18	12.9	5	3.6		33	23.6	31	22.1	20	14.3	7	5.0	
4	14	10.1	5	3.6	0	0.0	0	0.0		7	5.0	9	6.4	3	2.1	0	0.0	
N-stage									0.292[b]									0.421[b]
0	49	38.0	15	11.6	16	12.4	2	1.6		29	22.3	37	28.5	12	9.2	4	3.1	
1	18	14.0	9	7.0	3	2.3	4	3.1		11	8.5	11	8.5	9	6.9	3	2.3	
2	10	7.8	2	1.6	1	0.8	0	0.0		7	5.4	3	2.3	4	3.1	0	0.0	
M-stage									0.140[a]									0.150[a]
0	73	52.1	20	14.3	15	10.7	5	3.6		43	30.5	49	34.8	18	12.8	4	2.8	
1	13	9.3	7	5.0	6	4.3	1	0.7		10	7.1	5	3.5	9	6.4	3	2.1	
Vascular invasion									0.602[a]									0.660[a]
No	19	24.7	4	5.2	12	15.6	1	1.3		14	18.2	10	13.0	10	13.0	2	2.6	
Yes	22	28.6	11	14.3	5	6.5	3	3.9		12	15.6	17	22.1	8	10.4	4	5.2	
KRAS									**0.049[a]**									0.673[a]
Wild type	52	38.5	14	10.4	10	7.4	1	0.7		28	20.6	34	25.0	14	10.3	2	1.5	
Mutated	31	23.0	11	8.1	11	8.1	5	3.7		22	16.2	20	14.7	11	8.1	5	3.7	
BRAF									0.989[a]									0.689[a]
Wild type	80	59.3	24	17.8	20	14.8	6	4.4		49	36.0	51	37.5	24	17.6	7	5.1	
Mutated	3	2.2	1	0.7	1	0.7	0	0.0		1	0.7	3	2.2	1	0.7	0	0.0	
Differentiation									0.190[a]									0.078[a]
Low grade	10	7.2	7	5.0	5	3.6	0	0.0		6	4.3	8	5.7	7	5.0	2	1.4	
High grade	75	54.0	20	14.4	16	11.5	6	4.3		47	33.6	45	32.1	20	14.3	5	3.6	
Microsatellite stability									0.688[a]									0.731[a]
Stable	78	59.1	26	19.7	19	14.4	5	3.8		49	36.8	49	36.8	26	19.5	5	3.8	
Unstable	2	1.5	1	0.8	1	0.8	0	0.0		1	0.8	2	1.5	1	0.8	0	0.0	

Abbreviations: COMP, cartilage oligomeric matrix protein; The bold indicates *p*-values <0.05, [a] Calculated with Mann–Whitney U two-tailed exact *p*-value. [b] Calculated with Kruskal-Wallis exact *p*-value.

Table S3. COMP expression in the rectum and correlations with the clinical characteristics and associations of the patients.

Factor \ Score	Cancer cells 0		1		2		3		***p*-value**	Stroma 0		1		2		3		***p*-value**
All (N=208)	N	%	N	%	N	%	N	%		N	%	N	%	N	%	N	%	
Age at diagnosis									0.133[b]									0.102[b]
<50	1	0.5	0	0.0	0	0.0	0	0.0		1	0.5	0	0.0	0	0.0	0	0.0	
50-70	56	28.3	29	14.6	11	5.6	8	4.0		34	17.2	43	21.7	16	8.1	11	5.6	
>70	62	31.3	19	9.6	9	4.5	3	1.5		40	20.2	37	18.7	11	5.6	5	2.5	
Sex									0.929[a]									0.191[a]
Female	56	28.3	29	14.6	8	4.0	3	1.5		40	20.2	38	19.2	13	6.6	5	2.5	
Male	63	31.8	19	9.6	12	6.1	8	4.0		35	17.7	42	21.2	14	7.1	11	5.6	
T-stage									**0.030[b]**									**0.005[b]**
1	10	5.4	7	3.8	0	0.0	0	0.0		10	5.4	5	2.7	2	1.1	0	0.0	
2	29	15.6	5	2.7	2	1.1	1	0.5		16	8.6	15	8.1	5	2.7	1	0.5	
3	69	37.1	28	15.1	15	8.1	6	3.2		40	21.5	52	28.0	15	8.1	11	5.9	
4	6	3.2	2	1.1	3	1.6	3	1.6		2	1.1	4	2.2	4	2.2	4	2.2	
N-stage									0.067[b]									**0.006[b]**
0	71	40.1	24	13.6	8	4.5	3	1.7		45	25.4	45	25.4	12	6.8	4	2.3	
1	23	13.0	10	5.6	5	2.8	5	2.8		10	5.6	19	10.7	6	3.4	8	4.5	
2	15	8.5	4	2.3	6	3.4	3	1.7		8	4.5	10	5.6	6	3.4	4	2.3	
M-stage									0.249[a]									0.218[a]
0	105	53.8	43	22.1	15	7.7	10	5.1		67	34.4	70	35.9	22	11.3	14	7.2	
1	11	5.6	5	2.6	5	2.6	1	0.5		6	3.1	9	4.6	5	2.6	2	1.0	
Vascular invasion									0.129[a]									0.138[a]
No	40	34.8	15	13.0	5	4.3	2	1.7		20	17.4	32	27.8	7	6.1	3	2.6	
Yes	29	25.2	10	8.7	8	7.0	6	5.2		15	13.0	21	18.3	9	7.8	8	7.0	
KRAS									0.778[a]									0.528[a]
Wild type	78	41.5	33	17.6	10	5.3	7	3.7		52	27.7	49	26.1	17	9.0	10	5.3	
Mutated	36	19.1	13	6.9	9	4.8	2	1.1		21	11.2	25	13.3	10	5.3	4	2.1	
BRAF									0.367[a]									0.604[a]
Wild type	111	59.4	46	24.6	19	10.2	9	4.8		71	38.0	73	39.0	27	14.4	14	7.5	
Mutated	2	1.1	0	0.0	0	0.0	0	0.0		1	0.5	1	0.5	0	0.0	0	0.0	
Differentiation									0.051[a]									0.143[a]
Low grade	12	6.2	6	3.1	5	2.6	3	1.5		7	3.6	11	5.6	4	2.1	4	2.1	
High grade	106	54.4	40	20.5	15	7.7	8	4.1		66	33.8	68	34.9	23	11.8	12	6.2	
Microsatellite stability									0.643[a]									0.737[a]
Stable	105	57.7	44	24.2	20	11.0	10	5.5		68	37.4	70	38.5	26	14.3	15	8.2	
Unstable	2	1.1	1	0.5	0	0.0	0	0.0		1	0.5	2	1.1	0	0.0	0	0.0	

Abbreviations: COMP, cartilage oligomeric matrix protein; The bold indicates *p*-values <0.05, [a] Calculated with Mann–Whitney U two-tailed exact *p*-value. [b] Calculated with Kruskal-Wallis exact *p*-value.

Table S4 Cox multivariable COMP analyses in relation to disease progression markers and the anatomical site.

	**Right Colon**							
**Survival**	**Cancer cells**				**Stroma**			
**Variable**	**HR**	**95% CI**		***p*-value**	**HR**	**95% CI**		***p*-value**
COMP	0.719	0.494	1.048	0.086	0.945	0.705	1.268	0.706
BRAF	1.115	0.562	2.215	0.755	1.228	0.619	2.436	0.558
KRAS	0.974	0.496	1.911	0.939	1.057	0.535	2.088	0.873
Differentiation	1.584	0.875	2.867	0.129	1.595	0.878	2.899	0.125
T-stage	1.067	0.665	1.712	0.788	1.039	0.648	1.667	0.874
N-stage	1.299	0.840	2.009	0.240	1.281	0.827	1.985	0.268
M-stage	10.765	4.672	24.803	**<0.001**	9.802	4.336	22.161	**<0.001**
Vascular invasion	0.729	0.372	1.428	0.357	0.720	0.363	1.428	0.347
	**Left Colon**							
COMP	1.959	1.290	2.976	**0.002**	1.451	0.969	2.174	0.071
BRAF	1.818	0.371	8.908	0.461	1.509	0.308	7.408	0.612
KRAS	1.407	0.628	3.156	0.407	1.529	0.633	3.694	0.345
Differentiation	1.717	0.681	4.330	0.252	1.223	0.491	3.050	0.666
T-stage	2.201	0.913	5.308	0.079	1.474	0.673	3.227	0.332
N-stage	2.357	1.303	4.265	**0.005**	2.048	1.200	3.495	**0.009**
M-stage	6.963	2.951	16.428	**<0.001**	5.294	2.198	12.752	**0.000**
Vascular invasion	0.869	0.355	2.126	0.758	1.164	0.496	2.731	0.727
	**Rectum**							
COMP	1.241	0.942	1.636	0.125	1.447	1.079	1.940	**0.014**
BRAF								
KRAS	1.180	0.663	2.101	0.573	1.181	0.662	2.108	0.573
Differentiation	1.197	0.598	2.399	0.612	1.218	0.610	2.433	0.577
T-stage	2.292	1.395	3.765	**0.001**	2.266	1.411	3.640	**0.001**
N-stage	2.282	1.527	3.412	**<0.001**	2.234	1.496	3.335	**<0.001**
M-stage	1.352	0.571	3.204	0.493	1.326	0.568	3.095	0.514
Vascular invasion	2.564	1.362	4.827	**0.004**	2.478	1.322	4.646	**0.005**

Abbreviations: COMP, cartilage oligomeric matrix protein. The bold indicates *p*-values <0.05.

Table S5. Evaluation of COMP and PD-L1 associated expression categorized according to the anatomical site of the primary tumor.

	Right Colon									
	Cancer cells					Stroma				
	COMP Low		COMP High		*p*-value	COMP Low		COMP High		*p*-value
	N	(%)	N	(%)		N	(%)	N	(%)	
**PD-L1 immune cells**					0.065					0.073
0-9%	58	30.4	15	7.9		45	23.6	27	14.1	
10-49%	61	31.9	7	3.7		55	28.8	13	6.8	
50-100%	43	22.5	7	3.7		43	22.5	8	4.2	
**PD-L1 cancer cells**					0.200					**0.046**
<1%	108	56.5	20	10.5		90	47.1	37	19.4	
1-4%	19	9.9	2	1.0		18	9.4	3	1.6	
5-9%	7	3.7	5	2.6		8	4.2	5	2.6	
10-49%	9	4.7	0	0.0		9	4.7	0	0.0	
50-100%	19	9.9	2	1.0		18	9.4	3	1.6	
	**Left Colon**									
**PD-L1 immune cells**					0.225					0.185
0-9%	54	38.8	17	12.2		51	36.4	21	15.0	
10-49%	35	25.2	7	5.0		31	22.1	11	7.9	
50-100%	23	16.5	3	2.2		24	17.1	2	1.4	
**PD-L1 cancer cells**					0.739					0.810
<1%	99	71.2	25	18.0		93	66.4	32	22.9	
1-4%	9	6.5	2	1.4		9	6.4	2	1.4	
5-9%	2	1.4	0	0.0		2	1.4	0	0.0	
10-49%	2	1.4	0	0.0		2	1.4	0	0.0	
50-100%	0	0.0	0	0.0		0	0.0	0	0.0	
	**Rectum**									
**PD-L1 immune cells**					**0.022**					**<0.001**
0-9%	62	32.6	22	11.6		56	29.5	28	14.7	
10-49%	63	33.2	7	3.7		60	31.6	10	5.3	
50-100%	35	18.4	1	.5		34	17.9	2	1.1	
**PD-L1 cancer cells**					0.833					0.939
<1%	137	72.1	28	14.7		130	68.4	35	18.4	
1-4%	16	8.4	2	1.1		14	7.4	4	2.1	
5-9%	2	1.1	0	0.0		1	.5	1	.5	
10-49%	2	1.1	0	0.0		2	1.1	0	0.0	
50-100%	3	1.6	0	0.0		3	1.6	0	0.0	

Abbreviations: COMP, cartilage oligomeric matrix protein; PD-L1, programmed death-ligand 1; PD-1, Programmed cell death protein. Calculated with Kruskal-Wallis exact *p*-value. The bold indicates *p*-values <0.05

Table S6 Cox multivariable analyses of COMP expression with covariables related to the infiltrating immune cells stratified according to the anatomical site of the primary tumor.

	**Right Colon**							
**Survival**	**Cancer cells**				**Stroma**			
**Variable**	**HR**	**95% CI**		***p*-value**	**HR**	**95% CI**		***p*-value**
COMP	0.981	0.762	1.264	0.884	1.171	0.931	1.473	0.178
PD-L1 immune cells	0.673	0.494	0.916	**0.012**	0.718	0.525	0.983	**0.039**
PD-L1 cancer cells	1.113	0.946	1.308	0.197	1.144	0.970	1.348	0.109
$CD3^+$	0.583	0.340	0.999	**0.050**	0.588	0.344	1.004	0.052
$CD8^+$	1.379	0.806	2.359	0.242	1.389	0.813	2.373	0.230
$FoxP3^+$	0.961	0.634	1.456	0.851	0.989	0.654	1.496	0.958
$CD68^+$	0.577	0.362	0.919	**0.021**	0.550	0.347	0.872	**0.011**
$CD56^+$	0.641	0.414	0.992	**0.046**	0.687	0.441	1.071	0.098
$CD163^+$	2.046	1.278	3.277	**0.003**	1.872	1.131	3.097	**0.015**
	**Left Colon**							
COMP	1.277	0.977	1.670	0.073	1.598	1.148	2.225	**0.005**
PDL-1 immune cells	0.390	0.228	0.668	**0.001**	0.431	0.255	0.728	**0.002**
PDL-1 cancer cells	1.762	0.938	3.312	0.078	1.893	1.007	3.559	**0.047**
$CD3^+$	0.692	0.330	1.449	0.329	0.764	0.370	1.578	0.468
$CD8^+$	1.150	0.528	2.507	0.724	1.086	0.516	2.285	0.827
$FoxP3^+$	0.652	0.358	1.185	0.160	0.579	0.318	1.055	0.074
$CD68^+$	1.619	0.867	3.022	0.130	1.446	0.766	2.730	0.256
$CD56^+$	0.862	0.504	1.476	0.589	0.975	0.559	1.700	0.929
$CD163^+$	1.471	0.788	2.744	0.226	1.176	0.628	2.202	0.613
	**Rectum**							
COMP	1.359	1.062	1.740	**0.015**	1.370	1.072	1.751	**0.012**
PD-L1 immune cells	0.783	0.557	1.100	0.158	0.835	0.584	1.193	0.321
PD-L1 cancer cells	0.383	0.168	0.871	**0.022**	0.361	0.156	0.837	**0.018**
$CD3^+$	0.964	0.548	1.694	0.898	0.977	0.550	1.734	0.936
$CD8^+$	1.379	0.807	2.356	0.239	1.253	0.726	2.164	0.418
$FoxP3^+$	0.597	0.377	0.945	**0.028**	0.648	0.406	1.035	0.069
$CD68^+$	0.625	0.387	1.010	0.055	0.651	0.407	1.040	0.073
$CD56^+$	1.155	0.723	1.844	0.548	1.090	0.694	1.712	0.707
$CD163^+$	2.130	1.288	3.525	**0.003**	1.969	1.192	3.251	**0.008**

Abbreviations: COMP, cartilage oligomeric matrix protein. The bold indicates *p*-values <0.05.
